# Changes in Loneliness, Social Isolation, and Social Support: A Gender‐Disaggregated Analysis of Their Associations With Dementia and Cognitive Decline in Older Adults

**DOI:** 10.1002/gps.70065

**Published:** 2025-03-05

**Authors:** Htet Lin Htun, Achamyeleh Birhanu Teshale, Haoxiong Sun, Joanne Ryan, Alice J. Owen, Robyn L. Woods, Raj C. Shah, Trevor T.‐J. Chong, Rosanne Freak‐Poli

**Affiliations:** ^1^ School of Public Health and Preventive Medicine Monash University Melbourne Australia; ^2^ Department of Epidemiology and Biostatistics Institute of Public Health College of Medicine and Health Sciences University of Gondar Gondar Ethiopia; ^3^ School of Clinical Sciences at Monash Health Monash University Melbourne Australia; ^4^ Department of Family and Preventive Medicine and the Rush Alzheimer's Disease Center Rush University Medical Center Chicago Illinois USA; ^5^ Turner Institute for Brain and Mental Health School of Psychological Sciences Monash University Melbourne Australia; ^6^ Department of Neurology Alfred Health Melbourne Australia; ^7^ Department of Clinical Neurosciences St Vincent's Hospital Melbourne Australia

**Keywords:** cognitive decline, cognitive dysfunction, dementia, loneliness, social determinants of health, social isolation, social support

## Abstract

**Objectives:**

Limited evidence exists on the gender‐specific impact of changes in loneliness, social isolation, and social support on dementia risk. We examined these changes and their relationships with dementia and cognitive decline.

**Methods:**

Data from over 12,000 community‐dwelling Australians aged 70+ years without significant cognitive impairment at enrolment were analysed. Loneliness, social isolation, and social support were self‐reported at baseline and ∼2 years later (social isolation and social support) or ∼3 years later (loneliness), classified as never, transient, incident, or persistent. Dementia diagnosis followed *DSM‐IV* criteria, adjudicated by an expert panel. Gender‐disaggregated Cox proportional hazards regressions were conducted, adjusting for age and other dementia risk factors.

**Results:**

At baseline, participants were aged 70–95 years (mean: 75.2 ± 4.3), with 54% being women. Overall, 81.1% of men and 71.7% of women reported never feeling lonely at baseline, while transient, incident, and persistent loneliness were experienced by 4.9%, 8.4%, and 5.5% of men and 8.5%, 11.6%, and 8.3% of women, respectively. Over a median 8‐year follow‐up, incident loneliness in men (HR: 1.52, 95% CI: 1.08–2.13) and persistent loneliness in women (HR: 2.14, 95% CI: 1.55–2.97) were associated with a greater dementia risk, compared to those who were never lonely. No increased risk was observed for transient loneliness. Despite the remarkably low prevalence of social isolation and poor social support in this initially healthy cohort, both were associated with cognitive decline (secondary outcome) but not with dementia risk.

**Conclusion:**

Persistent loneliness in people aged 70+, especially in women, was associated with a higher risk of dementia and cognitive decline.

**Summary**

- All patterns of loneliness (transient, incident, persistent) were more prevalent in women than in men.
- Incident loneliness was associated with a higher risk of dementia in men.
- Persistent loneliness was associated with a higher risk of dementia in women.
- Changes in social isolation and low social support were associated with a greater risk of cognitive decline in both men and women, but not with dementia.

## 1 Introduction

Dementia poses a significant global health challenge, particularly within an ageing population. In 2019, 57.4 million people were living with dementia worldwide, a figure expected to reach 152.8 million by 2050 [1]. To reduce the disease burden, it is important to better understand modifiable risk factors for implementing early prevention strategies. One such factor of interest is social connection [2], a broad term describing a continuum that includes the size and diversity of an individual's social network and roles, the functions these relationships serve, and their positive or negative qualities [3]. Social connection is commonly assessed through loneliness, social isolation, and social support [4]. Loneliness is defined as a subjective distressing experience that results from perceived isolation or inadequate meaningful connections [3]. Social isolation refers to an objective state of having few social relationships, social roles, group memberships, and infrequent social interactions [3]. Social support reflects the perceived or actual availability of informational, tangible, and emotional resources from others [3]. They are particularly relevant among older people, as retirement, bereavement, and physical decline can reduce opportunities for social engagement [5, 6, 7].

Numerous systematic reviews demonstrate the association between various aspects of poor social connections and an increased risk of dementia and cognitive decline [8, 9, 10]. We previously investigated the relationship between cognitive outcomes and different social connection measures assessed at baseline, including loneliness, social isolation, and low social support, in a cohort of community‐dwelling healthy older people [11]. No associations were found between these measures and incident dementia over a 4.4‐year median follow‐up, nor with cognitive decline over a 3.1‐year median follow‐up. We noted as a limitation that a longer follow‐up period may be necessary, given the prolonged preclinical stage of dementia and slow progression of cognitive decline, which may require additional time to observe clinical symptoms in an initially healthy cohort [11].

Similar to our study, many others have mainly focused on single timepoint measurements. Since most individuals experience feelings of isolation or loneliness at various points in their lives, a single measurement may not fully capture the influence of social connection on health outcomes. Growing evidence now highlights the importance of assessing persistent social (dis)connection over time [12, 13]. Increasingly, researchers are examining longitudinal changes in social connection (typically across two timepoints) and their associations with health outcomes, including mortality, cardiovascular disease, stroke, and functional disability [14, 15, 16]. For instance, increased social isolation over time was associated with a 10% higher risk of mortality compared to a stable level [14].

While changes in social connection may influence both the progression and reversal of cognitive outcomes, research on these changes remains limited, leaving a knowledge gap. Gender‐specific findings are particularly scarce, despite well‐documented gender differences in social connections and dementia risk [17, 18]. To address these gaps, we aimed to examine the gender‐specific associations between changes in loneliness, social isolation, social support and the risk of dementia in relatively healthy community‐dwelling adults aged 70+, with cognitive decline as a secondary outcome. Additionally, we revisited prior analyses on baseline (single timepoint) social connection measures and their associations with dementia and cognitive decline [11], using an updated dataset with a longer follow‐up (median now exceeding 8 years).

## 2 Methods

### 2.1 Participants

**FIGURE 1:**
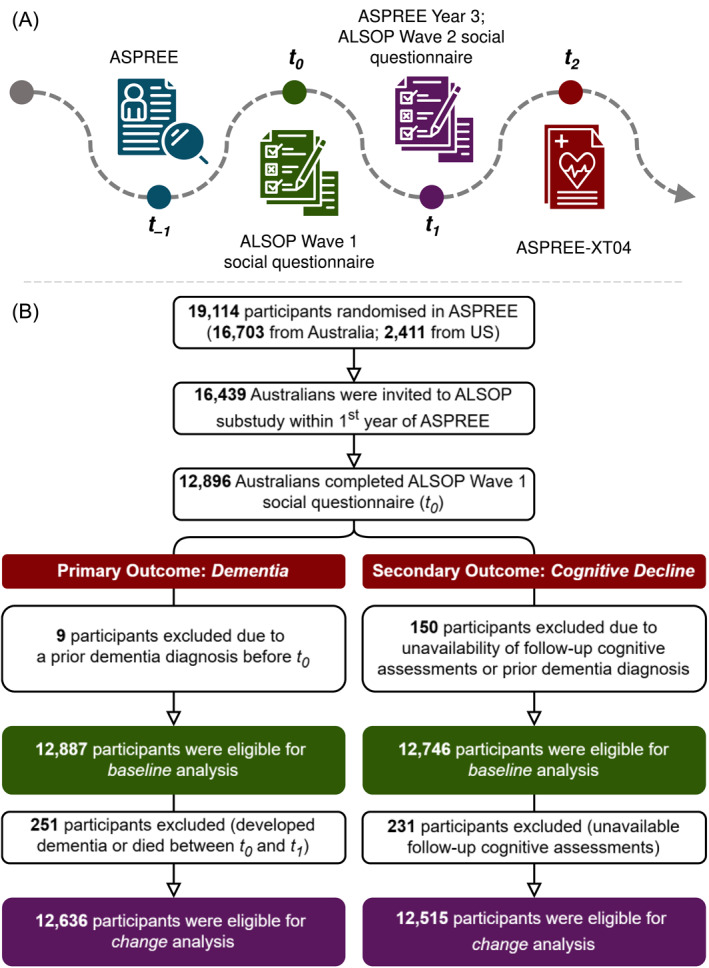
(A) Study timeline, and (B) Flowchart of participant selection. Loneliness was measured at the ASPREE enrolment (*t*_−1_) and ASPREE Year 3 visit (*t*_1_) (which generally coincided with the ALSOP Wave 2 timeline). Social isolation and social support were assessed using the ALSOP social questionnaires at Wave 1 (*t*_0_) and Wave 2 (*t*_1_). The median duration between *t*_0_ and *t*_1_ was 2.4 years, while the median duration between *t*_−1_ and *t*_1_ was ∼3 years. Outcomes were obtained using ASPREE‐XT04 (*t*_2_) dataset. Median follow‐up durations varied: 8.4 years for dementia (from *t*_−1_ ASPREE), 7.6 years for dementia (from *t*_0_ ALSOP Wave 1), 7.6 years for cognitive decline (from *t*_−1_ ASPREE), and 7 years for cognitive decline (from *t*_0_ ALSOP Wave 1).

This cohort study used data from the ASPirin in Reducing Events in the Elderly (ASPREE) trial [19], its observational extension (ASPREE‐XT) [20], and the ASPREE Longitudinal Study of Older Persons (ALSOP) substudy [21] (Figure 1A). ASPREE was a double‐blind, randomised, placebo‐controlled trial that examined the effects of daily 100 mg aspirin on various health outcomes. Between March 2010 and December 2014 (*t*_–1_), ASPREE enrolled 19,114 participants from Australia (87%) and the US (13%) through their primary healthcare providers. Participants were generally healthy individuals aged 70+ years (or 65+ for African American or Hispanic participants in the US), without dementia, cognitive impairment (i.e., Modified Mini‐Mental State examination [3MS] score < 78/100), cardiovascular diseases, or independence‐limiting physical disability. Analysis at the conclusion of the ASPREE intervention phase (4.7‐year median follow‐up) found no evidence that aspirin reduced dementia risk [22]. Therefore, intervention arm assignment was not adjusted in the present study. After the ASPREE intervention, participants were invited to join the ASPREE‐XT observational study, continuing annual follow‐ups to date [20].

Only Australian participants from the ASPREE trial were invited to ALSOP, which involved participant‐completed medical and social questionnaires. Wave 1 (*t*_0_) was administered within the first year of a participant's enrolment in ASPREE, followed by Wave 2 (*t*_1_) ∼2 years later. These questionnaires collected lifestyle, behavioural, and psychosocial data. Outcomes were obtained from the most recent ASPREE‐XT04 dataset, collected through the fourth annual visit of ASPREE‐XT (*t*_2_). After excluding individuals with prior dementia or missing follow‐up cognitive assessments, 12,636 participants remained for dementia and 12,515 for cognitive decline analyses (Figure 1B).

### 2.2 Measures

#### 2.2.1 Social (dis)connection

The measures, definitions, and cutoffs used in this study were based on prior studies within this cohort [11, 17, 18] (Supporting Information S1: Table S1).

1. *Loneliness* was assessed through a single question from 10‐item version of the Centre for Epidemiologic Studies Depression Scale (CES‐D‐10), which asked participants how often they felt lonely during the past week. Response options included *rarely or none of the time (* *<* *1 day), some or a little of the time (* *1–2* *days), occasionally or a moderate amount of the time (* *3–4* *days)* and *all of the time (* *5–7* *days).* Participants were classified as lonely if they reported feeling lonely for ≥ 1 day [17].
2. *Social isolation* was evaluated using the following questions. The first asked participants how many friends they see or hear from at least once a month, with response options ranging from *none* to *nine or more.* The second question assessed the frequency of engagement in community activities, including attending clubs/local organisations, places of worship, or educational classes, with options from *never* to *always.* Participants with fewer than five friends in monthly contact and engaging in community activities less than once a month were classified as socially isolated.
3. *Low social support* was assessed using two questions. The first assessed the number of friends and relatives with whom participants felt comfortable discussing private matters, with response options ranging from *none* to *nine or more.* The second question asked the number of friends and relatives whom participants felt close enough to call for help, using identical response options. Low social support was defined as having fewer than three friends or relatives in both questions.
4. As a secondary exposure, a *composite social disconnection* was defined by the presence of any one of the following: loneliness, social isolation, or low social support.

Social isolation and social support were measured at *t*_0_ and *t*_1_, while loneliness was assessed at *t*_–1_ and *t*_1_ (see Figure 1 footnote for details). Four change patterns were defined: ‘never’ (absent at both timepoints), ‘persistent’ (present at both timepoints), ‘transient’ (present at *t*_–1_/*t*_0_, absent at *t*_1_) and ‘incident’ (absent at *t*_–1_/*t*_0_, present at *t*_1_).

#### 2.2.2 Dementia and Cognitive Decline

A detailed report has been published elsewhere [22]. The cognitive battery included: (1) the 3MS examination for global cognition [23], (2) the Hopkins Verbal Learning Test‐Revised delayed recall task for episodic memory [24], (3) the single‐letter controlled oral word association test for executive function and verbal fluency [25], and (4) the Symbol Digit Modalities Test to assess psychomotor speed [26]. Participants underwent these assessments at baseline and at follow‐up visits in years 1, 3, 5, and during a final evaluation in 2017 as part of the ASPREE trial‐phase. Annual cognitive assessments continued into the ASPREE‐XT phase.

*Dementia* (primary outcome): Individuals suspected of having dementia (based on predefined triggers: a 3MS score < 78/100, a drop in age‐education adjusted predicted 3MS score of > 10.15 points from baseline, self‐reported cognitive issues, a clinician diagnosis of dementia, or prescription of cholinesterase inhibitors) were referred for further cognitive and functional assessments [22]. An adjudication committee reviewed these results and diagnosed dementia according to the *DSM‐IV* criteria.

*Cognitive decline* (secondary outcome): This was defined as a decline of > 1.5 standard deviations (SD) from an individual's baseline value on any of the four cognitive tests [22]. Participants with transient declines (e.g., > 1.5 SD drop at one follow‐up but later returned above this threshold) were excluded from this definition.

### 2.3 Statistical Analysis

Data were summarised using frequencies and percentages, mean with SD, or median with interquartile range (IQR). Bivariate analyses included Pearson's *χ* ^2^ test for categorical variables, and one‐way analysis of variance (ANOVA) or Kruskal‐Wallis test for continuous variables. We summarised overall characteristics of men and women, and patterns of changes in loneliness. Persistent social isolation and low social support were not summarised due to small sample sizes (ranging from 25 to 54; see Section 3), which limited statistical power and raised concerns about participant re‐identification from small cell counts.

Cox proportional hazards models, reporting hazard ratios (HR) and 95% confidence intervals (CI), were used to estimate the associations. The multivariable models adjusted for age, education, alcohol consumption, smoking, hypertension, diabetes, dyslipidaemia, hearing impairment, depressive symptoms, body mass index, and physical activity, with simultaneous adjustments for social isolation, low social support, and loneliness. To determine whether data were missing completely at random (MCAR), we conducted Little's MCAR test, which showed that the assumption was not met (*p*‐value < 0.05). Consequently, to avoid introducing selection bias from a complete‐case analysis, we first imputed missing data (< 5%) using *missRanger* function in R [27]. This method combines chained random forests and predictive mean matching to account for complex relationships between variables and generate more accurate imputations. A sensitivity analysis was conducted to address reverse causality (i.e., the potential influence of early subtle dementia symptoms on cognitive tests) by excluding individuals diagnosed with dementia during the initial three years of follow‐up. All statistical analyses were gender‐disaggregated (men and women, as no other gender identities were recorded) and performed using Stata/MP v.17 and R v.4.2.0. Statistical significance was set at a two‐tailed *p*‐value of < 0.05.

## 3 Results

**FIGURE 2:**
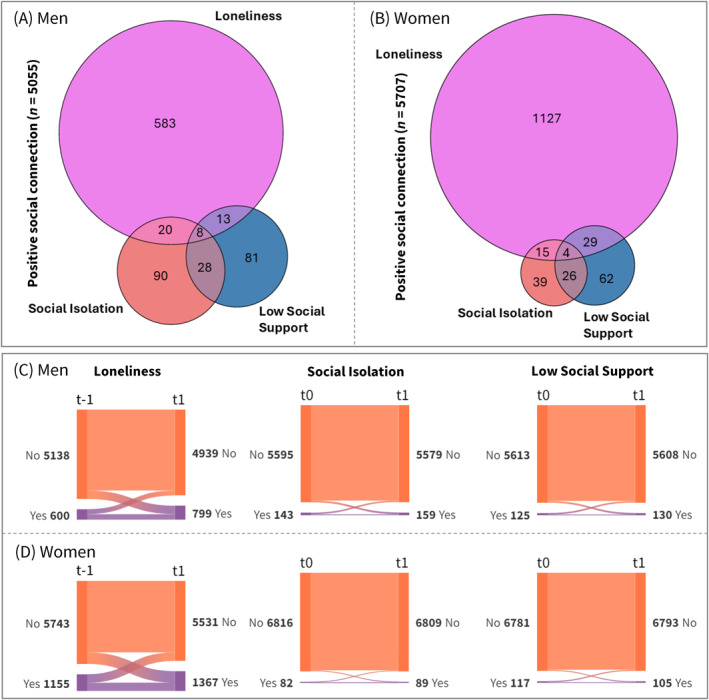
Distribution of loneliness, social isolation, and low social support at *t*_–1_/*t*_0_ for (A) men (*n* = 5878) and (B) women (*n* = 7009), along with changes in these measures between *t*_–1_/*t*_0_ and *t*_1_ for (C) men (*n* = 5738) and (D) women (*n* = 6898). The Venn and alluvial diagrams are proportionally scaled to reflect the sample size of each group. Larger circles in the Venn diagrams and larger nodes in the alluvial diagrams represent groups with bigger sample sizes. The correlations between these measures were low at baseline, with correlation coefficients ranging from 0.3 (between social isolation and low social support) to 0.03 (between social isolation and loneliness, and between loneliness and low social support).

We analysed data from > 12,000 older adults (range: 12,515–12,887), with 54.4% women (Figure 1 and Table 1). The participants were aged between 70 and 95 years (mean 75.2 ± 4.3), and > 98% identified as White/Caucasian. At baseline, majority of men (*n* = 5055, 86%) and women (*n* = 5707, 81%) were classified as socially connected. A higher percentage of men than women were socially isolated (2.5% *vs*. 1.2%) or low social support (2.2% *vs*. 1.7%), but they were less likely to report loneliness (10.6% *vs*. 16.8%). Additionally, more men than women reported feeling socially isolated and/or having low social support without feeling lonely (3.4% *vs*. 1.8%) (Figure 2A–2B).

**TABLE 1:** Characteristics of study participants.

Characteristics	Eligible participants (*n* = 12,887)^a^	
	Men (*n* = 5878)	Women (*n* = 7009)
Age, years		
Mean ± SD	75.1 ± 4.3	75.3 ± 4.3
Median (IQR)	73.9 (71.6–77.4)	74.1 (71.8–77.8)
Range	70.1–93.3	70.0–94.8
Ethnicity		
White/Caucasian	5789 (98.5)	6931 (98.9)
All other groups^b^	89 (1.5)	78 (1.1)
Education		
< 12 years	2658 (45.2)	3563 (50.8)
12–15 years	1526 (26.0)	1914 (27.3)
≥ 16 years	1694 (28.8)	1532 (21.9)
Alcohol consumption^c^		
Never	506 (8.6)	1505 (21.5)
Former	323 (5.5)	273 (3.9)
Moderate	2972 (50.6)	4025 (57.4)
Excessive	2077 (35.3)	1206 (17.2)
Current smoking	199 (3.4)	161 (2.3)
Hypertension^d^	4437 (75.5)	5142 (73.4)
Diabetes^e^	686 (11.7)	545 (7.8)
Dyslipidaemia^f^	3295 (56.1)	5390 (76.9)
Hearing impairment	3203 (54.5)	2638 (37.6)
CES‐D‐10 score^g^		
< 8	5444 (92.6)	6301 (89.9)
≥ 8	434 (7.4)	708 (10.1)
Body mass index, kg/m^2^		
Mean ± SD	27.9 ± 3.8	28.0 ± 5.0
Median (IQR)	27.5 (25.3–30.0)	27.3 (24.4–30.9)
Self‐reported physical activity intensity in a week		
Never/Rarely	72 (1.2)	114 (1.6)
No more than light	1474 (25.1)	2663 (38.0)
No more than moderate	3158 (53.7)	3361 (48.0)
Regular vigorous	1174 (20.0)	871 (12.4)

*Note:* Data are presented as *n* (*column* %) unless otherwise specified. Most characteristics were derived from ASPREE data at enrolment (*t*_−1_), except for *hearing impairment* and *physical activity*, which were obtained from ALSOP Wave 1 (*t*_0_).

Abbreviations: CES‐D‐10, 10‐item version of the Center for Epidemiologic Studies Depression Scale; IQR, interquartile range; SD, standard deviation.

^a^ The number of participants reported here is based on those eligible for the baseline social connection measures used to analyse dementia outcomes, representing the largest subset among the various subsamples, and the data presented use imputed values.

^b^ All other groups included Aboriginal/Torres Strait Islanders (*n* = 9), Native Hawaiian/Pacific Islander/Māori, Asian, American Indian, Black, more than one race and those whose race could not be determined. The racial categories used in this study reflect those established in the ASPREE study, which included participants from both the US and Australia. Although this analysis is restricted to the Australian population (drawn from ALSOP substudy), these categories were retained to maintain consistency with the original ASPREE framework and to provide a comprehensive overview of diversity within the sample.

^c^ Alcohol consumption was classified according to Australia's National Health and Medical Research Council guidelines. Moderate alcohol consumption comprises those who drink no more than 10 standard drinks a week AND no more than 4 standard drinks on any day. If these limits are exceeded, it is classified as excessive alcohol consumption.

^d^ Hypertension was defined as one or more of the following: prescription of antihypertensive medications, systolic blood pressure ≥ 140 mmHg, or diastolic blood pressure ≥90 mmHg.

^e^ Diabetes was defined as one or more of the following: self‐reported diabetes, prescription of glucose‐lowering medications, or a fasting blood sugar of ≥ 7.0 mmol/L (126 mg/dL).

^f^ Dyslipidaemia was defined as one or more of the following: prescription of cholesterol lowering medications, total serum cholesterol ≥ 5.5 mmol/L (212 mg/dL), or LDL‐cholesterol > 4.1 mmol/L (160 mg/dL).

^g^ Depressive symptoms were assessed using the 10‐item version of the Center for Epidemiologic Studies Depression Scale (CES‐D‐10). A total score of eight or higher was considered indicative of depression.

Persistent social isolation affected approximately 22% of men and 17% of women who reported social isolation, while persistent low social support affected 15% of men and 19% of women who reported low social support at either timepoints. Notably, more participants experienced new‐onset (incident) loneliness than those who were no longer lonely at *t*_1_ (transient). Therefore, total loneliness increased by 33% in men and 18% in women. Persistently lonely individuals represented about 29% of total loneliness reported by both men and women (Figure 2C–2D). They were generally older, had lower education, higher depressive symptoms, lower physical activity, and were more likely to live alone (Supporting Information S1: Table S2).

**FIGURE 3:**
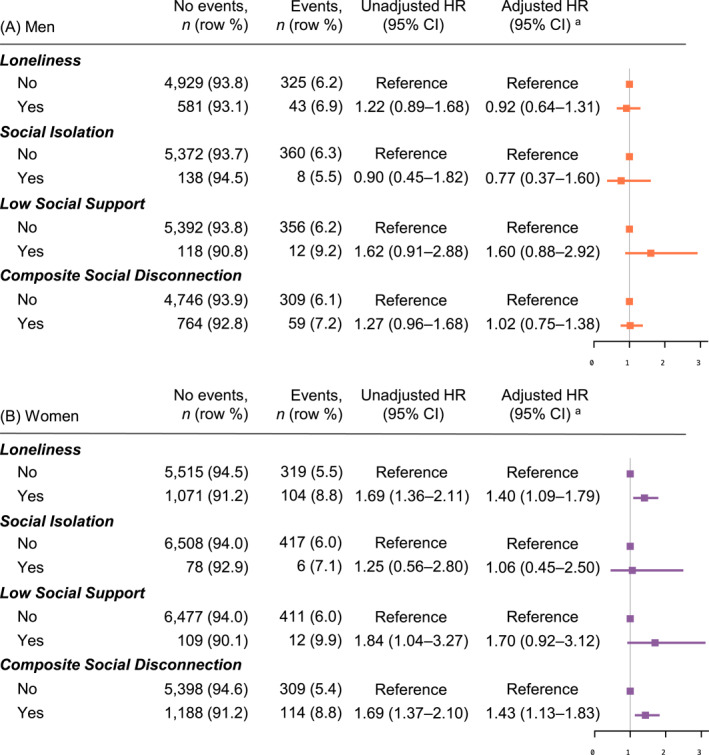
Association between baseline social connection measures (loneliness, social isolation, and low social support) and risk of dementia in (A) men (*n* = 5878) and (B) women (*n* = 7009). ^a^Adjusted for age, education, alcohol consumption, smoking, hypertension, diabetes, dyslipidaemia, hearing impairment, CES‐D‐10 depressive symptoms score, body mass index, and physical activity. Additionally, simultaneous adjustments for social isolation, low social support, and loneliness were performed, except when composite social disconnection was the exposure.

**FIGURE 4:**
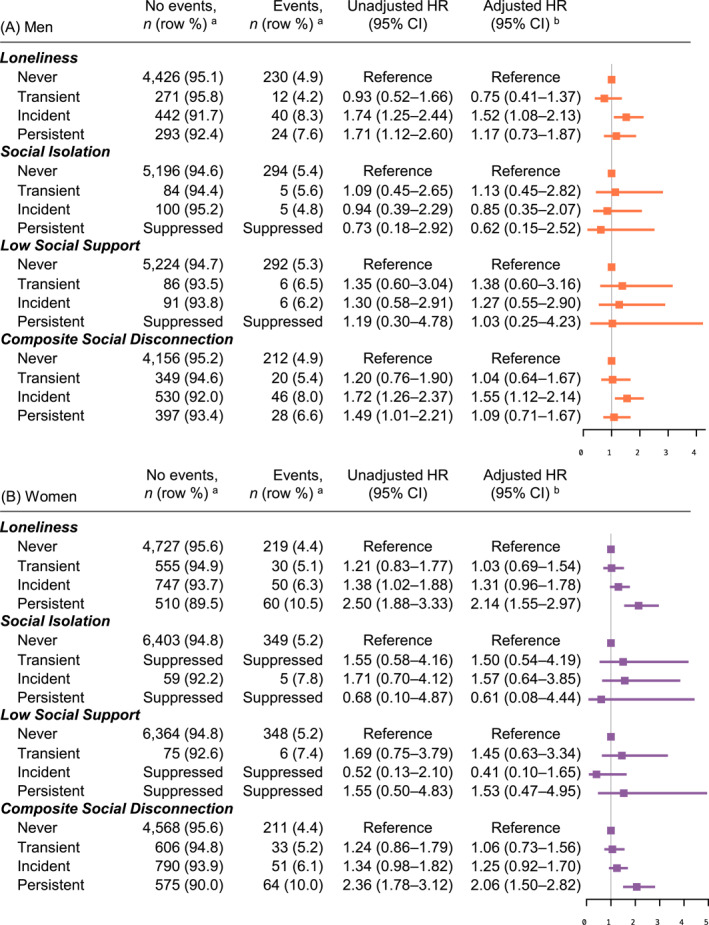
Association between change in social connection measures (loneliness, social isolation, and low social support) and risk of dementia in (A) men (*n* = 5738) and (B) women (*n* = 6898). ^a^Numbers are suppressed when the cell count is < 5. ^b^Adjusted for age, education, alcohol consumption, smoking, hypertension, diabetes, dyslipidaemia, hearing impairment, CES‐D‐10 depressive symptoms score, body mass index, and physical activity. Additionally, simultaneous adjustments for social isolation, low social support, and loneliness were performed, except when composite social disconnection was the exposure.

Dementia was diagnosed in 6.3% (*n* = 368/5878) of men and 6.0% (*n* = 423/7009) of women over 8‐year median follow‐up. Baseline loneliness was associated with a higher dementia risk in women (HR: 1.40, 95% CI: 1.09–1.79), while no association was observed in men (Figure 3). Incident and persistent loneliness were associated with an increased dementia risk in both men and women in unadjusted analyses. However, after adjusting for potential confounders, only incident loneliness in men (HR: 1.52, 95% CI: 1.08–2.13) and persistent loneliness in women (HR: 2.14, 95% CI: 1.55–2.97) remained associated with dementia, compared to never‐lonely individuals. No increased risk was observed for transient loneliness. Changes in social isolation and low social support showed no association with dementia (Figure 4). The effect estimates for composite social disconnection were similar to those for loneliness, as loneliness contributed the most to the composite measure. Sensitivity analyses excluding participants diagnosed with dementia within the first three years provided results consistent with the main findings (Supporting Information S1: Tables S3 and S4).

Similar associations were also found between loneliness and cognitive decline. In addition to changes in loneliness, social isolation in men and women, as well as low social support in women, were associated with an increased risk of cognitive decline (Supporting Information S1: Figures S1 and S2).

## 4 Discussion

### 4.1 Main Findings

In this cohort study of relatively healthy community‐dwelling Australians aged 70+ years, without major cognitive impairment at enrolment, we identified gender‐specific associations between social connection measures and the risk of dementia/cognitive decline over 8‐year median follow‐up. Baseline loneliness was associated with a 40% higher risk of dementia in women, but no association was found in men. Over time, compared to the never‐lonely group, incident loneliness in men was associated with a 52% increased risk, while persistent loneliness in women was associated with a 114% increased risk of dementia. Social isolation/support showed no association with dementia risk in either the baseline or change assessments. For cognitive decline, baseline social isolation was associated with a 43% higher risk in men, whereas baseline low social support was associated with a 47% increased risk in women. Regarding changes, incident loneliness was associated with a 20% increased risk of cognitive decline in men. In women, both incident and persistent loneliness were associated with higher risks, with 16% and 27% increases, respectively. Additionally, changes in social isolation for both men and women, along with low social support in women, were associated with an increased risk of cognitive decline. The following discussion primarily focuses on changes in social connection measures and dementia risk.

The literature on persistent loneliness and social isolation is still developing, with measurement inconsistencies contributing to variations in prevalence. A meta‐analysis of 17 studies published up to November 2023 found that persistent loneliness in older adults affected 16.3% (95% CI: 10.6%–21.9%) of men and 21.7% (95% CI: 16.1%–27.4%) of women, with a wide range (men: 6%–38%; women: 10%–45%) [12]. However, variations in items, collection timepoints, definitions, cutoffs used to assess loneliness complicate comparisons. An Australian study using the same item and cutoff as ours found that 8.8% of 1968 community‐based individuals aged 55+ from Newcastle experienced persistent loneliness across two timepoints [28]. Another Australian nationally representative survey reported a 13% prevalence of persistent loneliness and 4% for persistent social isolation, based on 10‐items Index of Social Support scale [13]. In our study, 5.5% of men and 8.3% of women reported feeling lonely on one or more days in the past week, considerably lower than the meta‐analysis and other Australian estimates [12, 13, 28], despite our lower loneliness threshold. This difference may reflect the relatively healthy, socioeconomically advantaged sample recruited for a clinical trial with strict inclusion criteria [19]. These variations also highlight the complex, context‐dependent nature of loneliness, shaped by cultural attitudes and societal norms [29]. Individual factors, such as personality and resilience, also influence susceptibility to loneliness [30].

Our study highlights an association between loneliness and the risk of dementia, with notable gender differences. While some studies align with our findings, others report varying results, likely due to differences in study sample and loneliness definitions [10, 28, 31, 32, 33]. For instance, the Framingham Heart Study found that persistent loneliness was associated with a higher dementia risk, transient loneliness with a lower risk, and incident loneliness showed no significant relationship. In contrast, an Australian study reported increased dementia risk for both transient and persistent loneliness [28]. Their transient group included individuals lonely at either of two timepoints, without differentiating between transient and incident loneliness [28]. However, these studies did not report gender‐stratified results, limiting direct comparisons. Additionally, our finding of null association between transient loneliness and adverse cognitive outcomes in either gender suggests that brief episodes of loneliness may not impose the same cognitive burden as persistent or newly developed loneliness in later life. This observation highlights the importance of assessing loneliness over time.

We advance the literature by providing the gendered perspective on changes in social connections and cognitive outcomes in older people. For women, persistent loneliness appears harmful for cognitive outcomes, suggesting a cumulative effect where ongoing social disconnection exacerbates cognitive vulnerability. Consistent with our findings, a US study among people aged 50+ found that a longer duration of loneliness was associated with a greater annual decline in memory scores in women compared to men [34]. This supports evidence indicating that women may be more sensitive to the long‐term health impacts of social disconnection, possibly due to differences in social networks/support and resilience factors [35, 36]. In contrast, new‐onset loneliness in later life was a risk factor for dementia in men, suggesting that the timing and sudden onset of loneliness may be more harmful than its duration. This indicates that men may experience adverse later‐life social changes in ways that significantly impact cognitive health. Abrupt shifts in social connectedness could challenge men's coping abilities [37], triggering stress responses or behavioural changes that elevate dementia risk [38]. Indeed, our previous analysis reported gender‐differences in the relationship between recent adverse life events and dementia risk [39].

Apart from changes in loneliness, social isolation and low social support had null findings with dementia, although they were associated with cognitive decline, partially corroborating prior findings [33]. This null association may reflect the low prevalence of social isolation and poor social support, reducing statistical power, coupled with our relatively healthy economically‐advantaged cohort. While the reasons for these differential associations remain unclear [40], it is plausible that these factors do not directly cause dementia pathology but instead contribute to an earlier onset of cognitive symptoms, such as cognitive impairments. Furthermore, gender differences are evident, as social support appears to influence the cognitive outcomes of women more than that of men. Our earlier studies suggest an optimal balance in perceived emotional support for women in relation to dementia risk (with 3–4 close relatives/friends being recommended) [17, 18, 41]. Notably, we found that a larger network for emotional support was associated with a 27% increased risk of dementia in women compared to an intermediate network [18]. Although this association might seem counterintuitive, our previous work offers theoretical insights [17, 18], drawing on frameworks such as the social complexity hypothesis, social contagion theory, and socioemotional selectivity theory, which suggest that a larger network may reflect diminished support quality or underlying socioemotional stressors. As the current study focuses on the social support changes on cognitive health outcomes, we refer readers to our earlier publications for further discussion.

### 4.2 Implications

Addressing persistent loneliness in older women and sudden‐onset loneliness in men may help reduce cognitive vulnerability, highlighting that these subpopulations would benefit most from interventions. A systematic review showed that psychological support, social support, and social access interventions effectively improve both the quantity and quality of social connections [42]. Similarly, a meta‐analysis of 27 studies examining individuals aged 65+ reported a moderate effect size (*d* = −0.47, 95% CI: −0.62, −0.32) for the effectiveness of non‐medical/pharmacological loneliness interventions [43]. Additionally, emerging healthcare models such as social prescribing, connecting individuals to community resources, offer further support, complementing traditional healthcare approaches while also considering the risks associated with hospital‐based care [44, 45, 46, 47, 48, 49].

### 4.3 Limitations and Strengths

We note several limitations. First, the transition from a clinical trial to an observational study may have introduced a healthy cohort effect, while our study population predominantly consisted of White and socioeconomically‐advantaged older Australians. This skewed representation may limit the generalisability. However, as more individuals reach older age in generally good health, our findings may become increasingly applicable. Additionally, it is plausible that the associations would be similar or even stronger in a more heterogeneous population with varying social connections and differing levels of health and wellbeing. Second, our study had a higher prevalence of positive social connections compared to other studies [12, 13, 28], likely reflecting the healthy cohort effect discussed earlier. The small number of affected individuals may have obscured the impact of social isolation and low social support on cognitive health, and we advise caution in interpreting these associations as their low prevalence may limit the generalisability of the findings. Further research in other community cohorts is required to validate these associations. Third, to address reverse causality, we conducted a sensitivity analysis by excluding participants diagnosed with dementia within the initial three years, although complete elimination remains challenging. Subtle prodromal symptoms of dementia may manifest prior to the full clinical presentation, given its extended preclinical phase [50]. Fourth, it is also possible that social isolation may modify the effect of loneliness on dementia, a hypothesis we were unable to explore further due to low prevalence in our study. This warrants investigation in future studies. Finally, while we used two timepoints of social connection measures to define patterns, consistent with prior studies [14, 15, 16, 28], this approach limits our ability to capture longitudinal trajectories fully. Future research should investigate these trajectories and their relationships with dementia using additional timepoints, such as the Wave 3 ALSOP data, when available.

The strength of this study is the prospective follow‐up of a large cohort of people without cognitive deficits at baseline. Regular standardised cognitive assessments by trained staff allowed for timely detection of cognitive decline, and dementia events were adjudicated by an expert panel, minimising misclassification and information biases. Distinguishing transient from incident loneliness allowed us to assess the impact of new‐onset social disconnection on cognitive outcomes. Furthermore, adjusting for each component of social connection theoretically isolates the impact of each factor while accounting for its multifaceted nature.

## 5 Conclusions

This study builds on our previous work [11] by examining changes in social connection measures across two timepoints with extended follow‐up. New‐onset loneliness in men and persistent loneliness in women were both associated with a greater risk of dementia among community‐dwelling adults aged 70+ years. Although limited statistical power restricted our ability to assess the association between changes in social isolation or social support on dementia risk, these factors were associated with cognitive decline. Early identification of poor social connections, particularly loneliness, and timely intervention to prevent its progression into a chronic state may help maintain cognitive resilience, while also delaying cognitive decline and dementia onset.

## Ethics Statement

All human participants of the ASPREE clinical trial and ALSOP substudy provided informed consent on participation. The data of the present secondary data‐analysis study were from a 5‐year ASPREE clinical trial and ALSOP substudy [Trial Registration: International Standard Randomized Controlled Trial Number Register (ISRCTN83772183) and ClinicalTrials.gov (NCT01038583)]. The ASPREE trial was approved by multiple Institutional Review Boards in Australia and the U.S. (https://aspree.org). ALSOP has been reviewed and approved by the Monash University Human Research Ethics Committee (Social ALSOP: CF11/1935–2011001094). The present study was approved by the Monash University Human Research Ethics Committee to conduct secondary data analysis.

## Conflicts of Interest

*Raj C. Shah* reported being the site principal investigator or sub‐investigator for Alzheimer's disease clinical trials for which his institution (Rush University Medical Centre) is compensated [Athira Pharma Inc., Edgewater NEXT, Eli Lilly & Co. Inc., and Genentech Inc.]. *Trevor T.‐J. Chong* reported receiving honoraria for lectures from Roche. All other authors declare no competing interests.

## Supporting information

Supporting Information S1

## Data Availability

All individual participant data underlying the results reported in this manuscript are available upon request to qualified researchers, subject to approval of the analyses by the Principal Investigators and adherence to a standard data sharing agreement. The data will be accessible through a secure web‐based data portal. Information regarding requests for data access is provided on the website (https://aspree.org).
