# Supplementary material for: Changes in Loneliness, Social Isolation, and Social Support: A Gender‐Disaggregated Analysis of Their Associations With Dementia and Cognitive Decline in Older Adults
Source: Int J Geriatr Psychiatry. 2025 Mar 5;40(3):e70065. doi: 10.1002/gps.70065 (PMC11882408; doi:10.1002/gps.70065)
Supplement: Supplementary file 1 — Supporting Information S1 [file GPS-40-e70065-s001.pdf]

Changes in Loneliness, Social Isolation, and Social Support: A Gender-Disaggregated Analysis of Their Associations With Dementia and Cognitive Decline in Older Adults

SUPPLEMENTAL MATERIALS

TABLE S1. Definitions of social connection measures assessed in the present study

TABLE S2. Baseline characteristic of men and women by pattern of changes in loneliness

TABLE S3. Association between baseline social connection measures (loneliness, social isolation, and low social support) and the risk of dementia in men and women, after excluding participants diagnosed with dementia within the first three years

TABLE S4. Association between change in social connection measures (loneliness, social isolation, and low social support) and the risk of dementia in men and women, after excluding participants diagnosed with dementia within the first three years

FIGURE S1. Association between baseline social connection measures (loneliness, social isolation, and low social support) and risk of cognitive decline in (A) men (*n* = 5,812) and (B) women (*n* = 6,934)

FIGURE S2. Association between change in social connection measures (loneliness, social isolation, and low social support) and risk of cognitive decline in (A) men (*n* = 5,687) and (B) women (*n* = 6,828)

TABLE S1. Definitions of social connection measures assessed in the present study

Outcomes	Definitions and Item-Response Options	Coding
Loneliness	*Question*: I felt lonely during the past week.<br>*Options*:<br>1. Rarely or none of the time (<1 day)<br>2. Some or a little of the time (1–2 days)<br>3. Occasionally or a moderate amount of time (3–4 days)<br>4. All of the time (5–7 days)<br>*Derived from*: 10-Item Center for Epidemiologic Studies Depression Scale (CES-D-10) (Cronbach's α = 0.86)	Binary:<br>0 = <1 day<br>1 = 1+ days
Social isolation	*Question*: Considering all your friends, including those who live in your neighbourhood, how many do you see or hear from at least once a month?<br>*Options*:<br>1. None<br>2. 1<br>3. 2<br>4. 3–4<br>5. 5–8<br>6. 9 or more<br>*Question*: How often do you engage the following community activities: (1) go to a club, local organisation, neighbourhood or other small groups; (2) go to a church, temple or other place of worship, or take part in related activities; and (3) attend an educational class?<br>*Options*:<br>1. Never<br>2. Rarely (less than once a month)<br>3. Sometimes (1–3 times a month)<br>4. Often (once a week or more)<br>5. Always (most days)<br>*Definition*: Social isolation was defined as having fewer than five friends seen or heard from in a month and engaging in community activities less than once a month, based on these two questions.<br>*Derived from*: 6-Item Lubben Social Network Scale (Cronbach's α = 0.83) and 10-Item Duke Social Support Index (Cronbach's α = 0.77)	Binary:<br>0 = No<br>1 = Yes

Low social support	*Question*: How many friends and relatives do you feel at ease with, that you can talk to about private matters?<br>*Options*:<br>1. None<br>2. 1<br>3. 2<br>4. 3–4<br>5. 5–8<br>6. 9 or more<br>*Question*: How many friends and relatives do you feel close to, such that you could call on them for help?<br>*Options*:<br>1. None<br>2. 1<br>3. 2<br>4. 3–4<br>5. 5–8<br>6. 9 or more<br>*Definition*: Low social support was defined as having fewer than three friends or relatives with whom one feels at ease discussing private matters and feels close enough to call for help, based on these two questions.<br>*Derived from*: 6-Item Lubben Social Network Scale (Cronbach's $\alpha = 0$ .83)	Binary:<br>0 = No<br>1 = Yes

TABLE S2. Baseline characteristic of men and women by pattern of changes in *loneliness*

Characteristics	Men (n = 5,738)				P_1	Women (n = 6,898)				P_2
	Never	Transient	Incident	Persistent		Never	Transient	Incident	Persistent	
Number (row %)	4,656 (81.1)	283 (4.9)	482 (8.4)	317 (5.5)	–	4,946 (71.7)	585 (8.5)	797 (11.6)	570 (8.3)	–
Age at enrolment, years										
Mean ± SD	74.9 ± 4.1	75.8 ± 4.8	75.6 ± 4.6	76.8 ± 5.2	<.001	75.1 ± 4.2	75.8 ± 4.7	75.5 ± 4.2	76.1 ± 4.7	<.001
Median (IQR)	73.6 (71.6–76.9)	74.3 (71.8–78.4)	74.4 (71.8–78.4)	75.5 (72.2–80.7)	<.001	73.9 (71.7–77.5)	74.6 (71.9–78.6)	74.5 (72.1–78.0)	75.0 (72.1–79.2)	<.001
Education										
< 12 years	2,062 (44.3)	141 (49.8)	223 (46.3)	160 (50.5)	.007	2,424 (49.0)	325 (55.6)	442 (55.5)	309 (54.2)	.001
12–15 years	1,202 (25.8)	62 (21.9)	139 (28.8)	88 (27.8)		1,404 (28.4)	135 (23.1)	200 (25.1)	142 (24.9)	
≥ 16 years	1,392 (29.9)	80 (28.3)	120 (24.9)	69 (21.8)		1,118 (22.6)	125 (21.4)	155 (19.4)	119 (20.9)	
Alcohol consumption										
Never	386 (8.3)	32 (11.3)	50 (10.4)	28 (8.8)	.243	1,050 (21.2)	121 (20.7)	184 (23.1)	124 (21.8)	.619
Former	243 (5.2)	18 (6.4)	30 (6.2)	15 (4.7)		182 (3.7)	20 (3.4)	32 (4.0)	28 (4.9)	
Moderate	2,394 (51.4)	136 (48.1)	233 (48.3)	146 (46.1)		2,880 (58.2)	344 (58.8)	433 (54.3)	318 (55.8)	
Excessive	1,633 (35.1)	97 (34.3)	169 (35.1)	128 (40.4)		834 (16.9)	100 (17.1)	148 (18.6)	100 (17.5)	
Current smoking	129 (2.8)	18 (6.4)	26 (5.4)	13 (4.1)	<.001	97 (2.0)	16 (2.7)	22 (2.8)	23 (4.0)	.010
Hypertension	3,519 (75.6)	215 (76.0)	353 (73.2)	243 (76.7)	.658	3,636 (73.5)	433 (74.0)	575 (72.2)	415 (72.8)	.831
Diabetes	520 (11.2)	41 (14.5)	55 (11.4)	46 (14.5)	.122	357 (7.2)	46 (7.9)	71 (8.9)	56 (9.8)	.075
Dyslipidaemia	2,645 (56.8)	154 (54.4)	260 (53.9)	166 (52.4)	.266	3,786 (76.6)	449 (76.8)	619 (77.7)	447 (78.4)	.718
Hearing impairment	2,484 (53.4)	166 (58.7)	286 (59.3)	194 (61.2)	.002	1,780 (36.0)	244 (41.7)	337 (42.3)	237 (41.6)	<.001
CES-D-10 score										
< 8	4,481 (96.2)	198 (70.0)	438 (90.9)	195 (61.5)	<.001	4,707 (95.2)	435 (74.4)	717 (90.0)	344 (60.4)	<.001
≥ 8	175 (3.8)	85 (30.0)	44 (9.1)	122 (38.5)		239 (4.8)	150 (25.6)	80 (10.0)	226 (39.7)	
Body mass index, kg/m^2										
Mean ± SD	27.9 ± 3.7	28.0 ± 4.1	28.2 ± 4.1	28.0 ± 4.0	.001	27.9 ± 4.9	27.8 ± 5.0	28.3 ± 5.3	28.5 ± 5.7	<.001
Median (IQR)	27.4 (25.3–29.9)	27.2 (25.0–30.4)	27.8 (25.6–30.5)	27.8 (25.4–29.7)	.559	27.2 (24.4–30.8)	27.0 (24.4–30.7)	27.6 (24.3–31.5)	27.7 (24.6–31.4)	.099
Physical activity intensity										
Never/Rarely	43 (0.9)	7 (2.5)	8 (1.7)	6 (1.9)	<.001	60 (1.2)	15 (2.6)	17 (2.1)	18 (3.2)	<.001
No more than light	1,104 (23.7)	90 (31.8)	129 (26.8)	114 (36.0)		1,833 (37.1)	218 (37.3)	314 (39.4)	254 (44.6)	
No more than moderate	2,543 (54.6)	141 (49.8)	256 (53.1)	140 (44.2)		2,411 (48.8)	284 (48.6)	370 (46.4)	250 (43.9)	
Regular vigorous	966 (20.8)	45 (15.9)	89 (18.5)	57 (18.0)		642 (13.0)	68 (11.6)	96 (12.1)	48 (8.4)	
Living alone	458 (9.8)	135 (47.7)	132 (27.4)	213 (67.2)	<.001	1,693 (34.2)	374 (63.9)	335 (42.0)	402 (70.5)	<.001

Data are presented as n (column %) unless otherwise specified. Most characteristics were derived from ASPREE baseline data, except for hearing impairment and physical activity, which were obtained from ALSOP Wave 1. P_1, statistical tests between pattern of changes in men; P_2, statistical tests between pattern of changes in women.

TABLE S3. Association between baseline social connection measures (loneliness, social isolation, and low social support) and the risk of dementia in men and women, after excluding participants diagnosed with dementia within the first three years

Social Connection Measures	Adjusted HR (95% CI) [a]
**Men (*n* = 5,821)**	
Loneliness	
No	Reference
Yes	0.90 (0.61 – 1.33)
Social Isolation	
No	Reference
Yes	0.85 (0.39 – 1.85)
Low Social Support	
No	Reference
Yes	1.58 (0.82 – 3.03)
**Women (*n* = 6,949)**	
Loneliness	
No	Reference
Yes	1.44 (1.10 – 1.88)
Social Isolation	
No	Reference
Yes	1.08 (0.43 – 2.74)
Low Social Support	
No	Reference
Yes	1.68 (0.87 – 3.27)

[a] adjusted for age, education, alcohol consumption, smoking, hypertension, diabetes, dyslipidaemia, hearing impairment, depressive symptoms, body mass index, and physical activity, with simultaneous adjustments for social isolation, low social support, and loneliness.

TABLE S4. Association between change in social connection measures (loneliness, social isolation, and low social support) and the risk of dementia in men and women, after excluding participants diagnosed with dementia within the first three years

Social Connection Measures	Adjusted HR (95% CI) [a]
**Men (*n* = 5,733)**	
Loneliness	
Never	Reference
Transient	0.77 (0.42 – 1.40)
Incident	1.56 (1.11 – 2.19)
Persistent	1.20 (0.75 – 1.91)
Social Isolation	
Never	Reference
Transient	1.14 (0.46 – 2.86)
Incident	0.86 (0.35 – 2.10)
Persistent	0.63 (0.15 – 2.57)
Low Social Support	
Never	Reference
Transient	1.40 (0.61 – 3.21)
Incident	1.29 (0.56 – 2.94)
Persistent	1.05 (0.25 – 4.31)
**Women (*n* = 6,894)**	
Loneliness	
Never	Reference
Transient	1.04 (0.70 – 1.55)
Incident	1.33 (0.97 – 1.81)
Persistent	2.16 (1.56 – 2.99)
Social Isolation	
Never	Reference
Transient	1.51 (0.54 – 4.22)
Incident	1.59 (0.65 – 3.90)
Persistent	0.61 (0.08 – 4.45)
Low Social Support	
Never	Reference
Transient	1.47 (0.64 – 3.38)
Incident	0.41 (0.10 – 1.66)
Persistent	1.55 (0.48 – 5.02)

[a] adjusted for age, education, alcohol consumption, smoking, hypertension, diabetes, dyslipidaemia, hearing impairment, depressive symptoms, body mass index, and physical activity, with simultaneous adjustments for social isolation, low social support, and loneliness.

(A) Men	No events, n (row %)	Events, n (row %)	Unadjusted HR (95% CI)	Adjusted HR (95% CI)[a]
Loneliness				
No	3,812 (73.4)	1,384 (26.6)	Reference	Reference
Yes	429 (69.6)	187 (30.4)	1.34 (1.15–1.56)	1.06 (0.89–1.26)
Social Isolation				
No	4,143 (73.1)	1,524 (26.9)	Reference	Reference
Yes	98 (67.6)	47 (32.4)	1.57 (1.18–2.10)	1.43 (1.06–1.94)
Low Social Support				
No	4,153 (73.1)	1,530 (26.9)	Reference	Reference
Yes	88 (68.2)	41 (31.8)	1.44 (1.06–1.97)	1.18 (0.86–1.63)
Composite Social Disconnection				
No	3,679 (73.6)	1,319 (26.4)	Reference	Reference
Yes	562 (69.0)	252 (31.0)	1.39 (1.21–1.59)	1.14 (0.99–1.33)

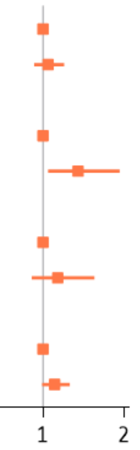

(B) Women	No events, n (row %)	Events, n (row %)	Unadjusted HR (95% CI)	Adjusted HR (95% CI)[a]
Loneliness				
No	4,180 (72.3)	1,598 (27.7)	Reference	Reference
Yes	795 (68.8)	361 (31.2)	1.25 (1.12–1.40)	1.07 (0.94–1.21)
Social Isolation				
No	4,922 (71.8)	1,932 (28.2)	Reference	Reference
Yes	53 (66.2)	27 (33.8)	1.23 (0.84–1.80)	1.00 (0.67–1.50)
Low Social Support				
No	4,901 (71.9)	1,916 (28.1)	Reference	Reference
Yes	74 (63.2)	43 (36.8)	1.53 (1.13–2.08)	1.47 (1.07–2.01)
Composite Social Disconnection				
No	4,100 (72.5)	1,554 (27.5)	Reference	Reference
Yes	875 (68.4)	405 (31.6)	1.27 (1.14–1.42)	1.10 (0.98–1.24)

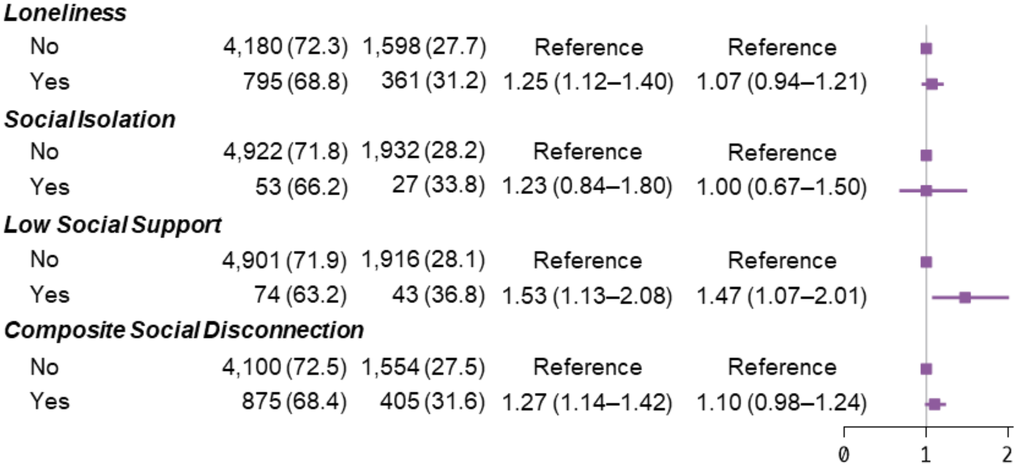

FIGURE S1. Association between baseline social connection measures (loneliness, social isolation, and low social support) and risk of cognitive decline in (A) men (n = 5,812) and (B) women (n = 6,934).

[a] adjusted for age, education, alcohol consumption, smoking, hypertension, diabetes, dyslipidaemia, hearing impairment, depressive symptoms, body mass index, and physical activity. Additionally, simultaneous adjustments for social isolation, low social support, and loneliness were performed, except when composite social disconnection was the exposure.

(A) Men	No events, n (row %)	Events, n (row %)	Unadjusted HR (95% CI)	Adjusted HR (95% CI) [a]
Loneliness				
Never	3,438 (74.5)	1,174 (25.5)	Reference	Reference
Transient	199 (71.6)	79 (28.4)	1.23 (0.98–1.54)	1.04 (0.82–1.33)
Incident	323 (67.2)	158 (32.6)	1.36 (1.51–1.61)	1.20 (1.01–1.42)
Persistent	211 (66.8)	105 (33.2)	1.60 (1.31–1.96)	1.19 (0.96–1.48)
Social Isolation				
Never	3,998 (72.3)	1,442 (26.5)	Reference	Reference
Transient	58 (65.2)	31 (34.8)	1.67 (1.17–2.39)	1.56 (1.08–2.26)
Incident	78 (74.3)	27 (25.7)	1.14 (0.78–1.67)	1.05 (0.71–1.54)
Persistent	37 (69.8)	16 (30.2)	1.60 (0.98–2.62)	1.43 (0.87–2.36)
Low Social Support				
Never	4,019 (73.5)	1,448 (26.5)	Reference	Reference
Transient	65 (71.4)	26 (28.6)	1.29 (0.87–1.90)	1.03 (0.69–1.53)
Incident	67 (69.8)	29 (30.2)	1.36 (0.94–1.97)	1.14 (0.78–1.66)
Persistent	20 (60.6)	13 (39.4)	1.89 (1.10–3.27)	1.58 (0.91–2.75)
Composite Social Disconnection				
Never	3,236 (74.8)	1,090 (25.2)	Reference	Reference
Transient	253 (69.5)	111 (30.5)	1.35 (1.11–1.64)	1.17 (0.95–1.43)
Incident	395 (68.8)	179 (31.2)	1.34 (1.15–1.64)	1.20 (1.02–1.41)
Persistent	287 (67.8)	136 (32.2)	1.59 (1.33–1.90)	1.25 (1.03–1.51)

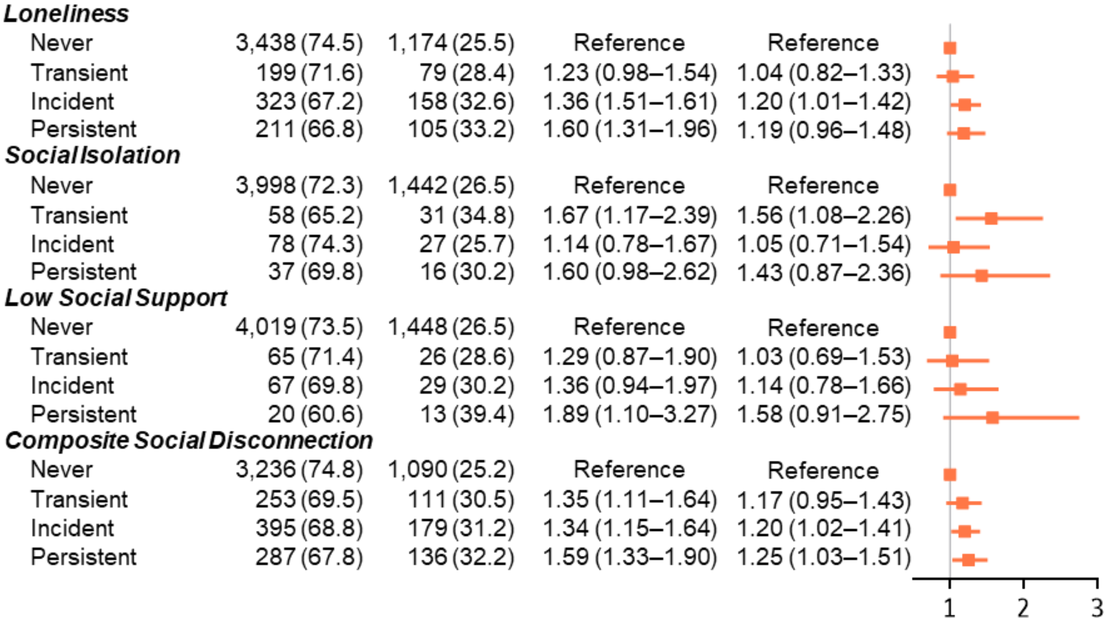

(B) Women	No events, n (row %)	Events, n (row %)	Unadjusted HR (95% CI)	Adjusted HR (95% CI) [a]
Loneliness				
Never	3,592 (73.4)	1,303 (26.6)	Reference	Reference
Transient	423 (73.9)	149 (26.1)	1.07 (0.91–1.27)	0.93 (0.78–1.11)
Incident	543 (68.2)	253 (31.8)	1.25 (1.09–1.43)	1.16 (1.01–1.33)
Persistent	367 (65.0)	198 (35.0)	1.50 (1.30–1.75)	1.27 (1.08–1.50)
Social Isolation				
Never	4,833 (72.3)	1,852 (27.7)	Reference	Reference
Transient	37 (68.5)	17 (31.5)	1.31 (0.81–2.10)	1.15 (0.70–1.87)
Incident	39 (60.9)	25 (39.1)	1.93 (1.30–2.87)	1.71 (1.14–2.56)
Persistent	16 (64.0)	9 (36.0)	1.08 (0.56–2.10)	0.87 (0.44–1.71)
Low Social Support				
Never	4,806 (72.3)	1,840 (27.7)	Reference	Reference
Transient	49 (63.6)	28 (36.4)	1.64 (1.13–2.38)	1.60 (1.10–2.34)
Incident	45 (65.2)	24 (34.8)	1.37 (0.92–2.05)	1.09 (0.73–1.64)
Persistent	25 (69.4)	11 (30.6)	1.11 (0.61–2.00)	1.00 (0.54–1.84)
Composite Social Disconnection				
Never	3,485 (73.5)	1,245 (26.3)	Reference	Reference
Transient	462 (74.0)	162 (26.0)	1.09 (0.93–1.29)	0.96 (0.81–1.14)
Incident	570 (67.9)	270 (32.1)	1.30 (1.14–1.48)	1.19 (1.04–1.36)
Persistent	408 (64.4)	226 (35.6)	1.52 (1.32–1.75)	1.31 (1.12–1.53)

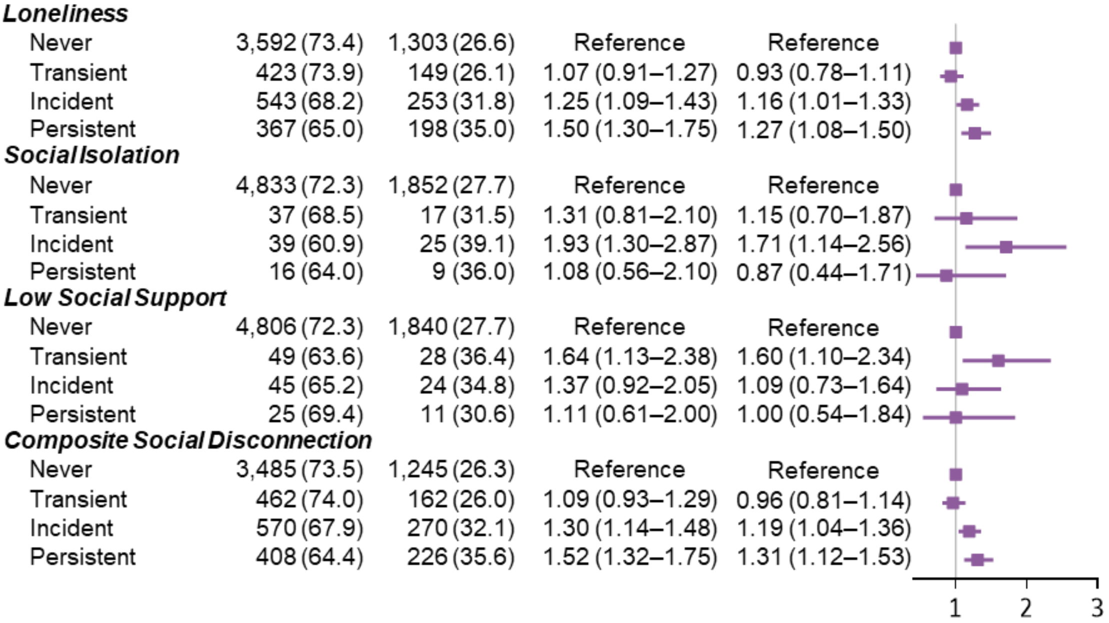

FIGURE S2. Association between change in social connection measures (loneliness, social isolation, and low social support) and risk of cognitive decline in (A) men (n = 5,687) and (B) women (n = 6,828).

[a] adjusted for age, education, alcohol consumption, smoking, hypertension, diabetes, dyslipidaemia, hearing impairment, depressive symptoms, body mass index, and physical activity. Additionally, simultaneous adjustments for social isolation, low social support, and loneliness were performed, except when composite social disconnection was the exposure.
